# Stratified profiling of azoospermia: Differentiating histological subtypes with seminal plasma metabolic signatures

**DOI:** 10.7555/JBR.39.20250294

**Published:** 2026-07-28

**Authors:** Jiachen Wang, Tianyi Song, Hongqi Fan, Mengyuan Zhu, Ying Yao, Laihua Li, Mingxi Liu, Minjian Chen, Jiahao Sha, Xiaoyu Yang, Yan Yuan

**Affiliations:** 1 State Key Laboratory of Reproductive Medicine and Offspring Health, Nanjing Medical University, Nanjing, Jiangsu 211166, China; 2 Department of Endocrinology, the First Affiliated Hospital of Nanjing Medical University, Nanjing Medical University, Nanjing, Jiangsu 210029, China; 3 Key Laboratory of Modern Toxicology of Ministry of Education, School of Public Health, Nanjing Medical University, Nanjing, Jiangsu 211166, China; 4 State Key Laboratory of Reproductive Medicine and Offspring Health, Clinical Center of Reproductive Medicine, the First Affiliated Hospital of Nanjing Medical University, Nanjing, Jiangsu 210029, China

**Keywords:** non-obstructive azoospermia, seminal plasma, metabolomics, diagnostic biomarkers

## Abstract

Non-obstructive azoospermia (NOA), characterized by impaired spermatogenesis and the complete absence of sperm in the ejaculate, represents one of the most severe forms of male infertility. Current approaches for definitive histological characterization and sperm retrieval often require invasive testicular procedures, underscoring the need for reliable, non-invasive methods for preoperative stratification. In the present study, we performed untargeted metabolomic profiling of human seminal plasma to identify biomarker panels capable of stratifying azoospermia subtypes through a stepwise approach. We identified distinct metabolite biomarker panels comparing NOA *vs.* obstructive azoospermia (OA), Sertoli cell-only syndrome *vs.* other NOA subtypes, and hypospermatogenesis *vs.* maturation arrest. Additionally, cross-species comparative analysis with high-resolution metabolomic data from purified mouse testicular cell populations implicated these biomarkers in specific germ cell stages and metabolic transitions during spermatogenesis. These findings provide a preliminary molecular framework for the non-invasive classification of azoospermia subtypes and warrant validation in larger independent cohorts.

## Introduction

Male infertility is a global health issue, affecting approximately 7% of the male population^[1]^. Among its most severe forms is non-obstructive azoospermia (NOA), a condition characterized by the absence of sperm in the ejaculate because of impaired spermatogenesis^[2–3]^. Clinical management of NOA is challenging, as definitive histological characterization and assessment of sperm retrieval ultimately rely on invasive testicular procedures. Testicular sperm extraction is not only costly and associated with potential complications, but its success also depends on the underlying testicular histology, which ranges from complete germ cell absence (Sertoli cell-only syndrome [SCO]) to varying degrees of spermatogenic arrest, such as maturation arrest (MA) and hypospermatogenesis (HS)^[4–7]^. A critical unmet need in reproductive medicine is the development of non-invasive methods to accurately predict the presence of sperm and differentiate these histological subtypes, thereby facilitating patient counseling and supporting preoperative decision-making^[8–9]^.

Seminal plasma, the complex extracellular fluid component of semen, functions not only as a transport medium for spermatozoa but also as a biochemical indicator of the male reproductive tract, particularly the testis and epididymis^[10–11]^. It is derived from secretions produced by various accessory glands, as well as from the seminiferous tubules and ductal systems, thereby encompassing a wide array of molecular signals indicative of spermatogenic activity. Because semen is readily obtainable through non-invasive means, seminal plasma represents an ideal and underutilized source of biomarkers for assessing testicular function and male fertility status^[12–14]^.

The present study employed metabolomics to elucidate the complex information contained in seminal plasma. This approach offers a unique analytical perspective, as small-molecule metabolites represent the ultimate functional output of cellular activity, thereby directly reflecting the phenotypic state of an organism. This makes metabolomics an ideal probe for studying highly metabolically active processes, such as spermatogenesis, one of the most metabolically demanding processes in humans^[15]^. The intricate germ cell proliferation, meiosis, and differentiation require a precisely orchestrated metabolic network. This includes the provision of lactate fuel from Sertoli cell glycolysis^[16]^, a crucial shift within germ cells from glycolysis to oxidative phosphorylation and fatty acid oxidation as they mature, and the activation of biosynthetic routes such as the pentose phosphate and one-carbon pathways to support cellular growth^[17]^. Consequently, any disruption in spermatogenesis is expected to leave a distinct metabolic signature^[18–19]^. The application of metabolomics in this field holds promise for more accurate diagnoses and personalized treatments for infertile men.

In the present study, we conducted a comprehensive metabolomic analysis of seminal plasma to address the pressing need for non-invasive diagnostic tools. Building on our previous identification of a metabolic signature that distinguished azoospermic patients from normospermic individuals^[20]^, we extended this approach to distinguish obstructive azoospermia (OA) from NOA cases and further differentiate major NOA subtypes. The present study followed a stratified diagnostic approach, mirroring the clinical pathway. As a foundational step, we first aimed to identify a candidate metabolic signature capable of distinguishing OA from NOA cases. Building upon this distinction, our primary and most clinically significant objective was to further stratify the heterogeneous NOA population by differentiating its key histological subtypes. Furthermore, to elucidate the biological underpinnings of our findings, we compared our human seminal plasma data with a metabolomic dataset derived from purified mouse spermatogenic and somatic testicular cells.

## Materials and methods

### Study subjects and seminal plasma samples

The human seminal plasma samples analyzed in the present study were obtained from a cohort of 80 azoospermia patients described in our previous publication^[20]^. All procedures were approved by the Institutional Review Board of the First Affiliated Hospital of Nanjing Medical University (Approval No. 2023-SZ-01) on 27 June 2023, and written informed consent was obtained from all participants. Briefly, participants were recruited from the Reproductive Medicine Center of the First Affiliated Hospital of Nanjing Medical University. The diagnosis of azoospermia was based on WHO guidelines, confirmed by the absence of sperm in the centrifuged pellet of at least two separate semen samples collected after 2–7 days of sexual abstinence. Exclusion criteria were comprehensive, including patients with genital tract infections, chromosomal abnormalities (including Y chromosome microdeletions), known systemic metabolic diseases (*e.g.*, diabetes), or other confounding factors affecting spermatogenesis. All seminal plasma samples were collected *via* masturbation and processed after liquefaction. Crucially, all samples were obtained before any surgical procedure, including testicular biopsy. The diagnostic criteria for OA and NOA were based on clinical parameters, including testicular volume, serum follicle-stimulating hormone levels, and physical examination, following established guidelines. Histological subtyping of NOA was performed on testicular biopsy specimens obtained via unilateral multi-site sampling, with typically three to six biopsy sites per patient. Tissue sections were stained with hematoxylin and eosin (H&E) and evaluated according to established criteria for human testicular histological evaluation^[5]^. NOA cases were classified as SCO, MA, or HS based on the predominant histological pattern observed across the examined seminiferous tubules.

### Metabolite extraction and ultra-high-performance liquid chromatography-quadrupole time-of-flight mass spectrometry (UHPLC-QTOF-MS) analysis

The metabolomic data matrix used in the present study was generated from the cohort detailed in our previous publication^[20]^. In the present work, new analyses focusing on previously unexamined aspects of this dataset were performed. The grouping strategy and technical pipelines applied here are novel and extend the scope of the original conclusions.

Briefly, seminal plasma was isolated from liquefied semen *via* multi-step centrifugation. Metabolites were then extracted using a 4∶1 methanol-to-water protein precipitation protocol. The resulting supernatant was dried, reconstituted, and analyzed by UHPLC-QTOF-MS.

Separation was performed on a Hypersil GOLD C18 column (100 mm × 2.1 mm, particle size 1.9 μm, Thermo Fisher Scientific, Waltham, MA, USA) with a gradient of acetonitrile and water, both containing 0.1% formic acid. Ionization was achieved using a heated electrospray ionization source in both positive and negative modes. Data were acquired in full-scan mode (m/z 70–1050) at a resolution of 70000. Metabolite identification was performed using TraceFinder software (v3.1, Thermo Fisher Scientific) by matching against an in-house library constructed from authentic chemical standards purchased from Sigma-Aldrich (St. Louis, MO, USA), Damas-beta Co., Ltd. (Basel, Switzerland), Adamas Reagent Co., Ltd. (Shanghai, China), and Aladdin Reagent Company (Shanghai, China). Metabolite annotation required simultaneous matching of accurate mass (tolerance ≤ 5 ppm) and retention time (tolerance ≤ 0.1 min), with MS/MS spectral confirmation whenever available. Metabolites matching authentic standards by accurate mass and retention time were considered Level 1 identifications when MS/MS spectra were also concordant. The final data matrix served as the input for all subsequent analyses.

### Data preprocessing

Prior to downstream multivariate and univariate analyses, we performed principal component analysis (PCA)-based diagnostics to identify potential outlier samples. Outlier detection was based on a combination of score distance (SD) and orthogonal distance (OD) thresholds, as implemented in the ropls package (v1.28.2)^[21]^. Samples exceeding the 95% confidence limits for these metrics were flagged as statistical outliers. A total of six samples—comprising three OA, two HS, and one MA—were identified as outliers. After confirming that these samples exhibited no clear pathological abnormalities based on histological reports and clinical metadata, we excluded them from further analysis to improve model robustness and biological interpretability.

Metabolites were filtered to ensure data quality: features with more than 80% zero values in all experimental groups were removed, as were features with no variance across all samples. Subsequent steps were performed to align with the MetaboAnalyst workflow^[22]^. Orthogonal partial least squares-discriminant analysis (OPLS-DA) was performed using the ropls package. Model quality was assessed *via* 200-permutation testing with intercept analysis.

### Biomarker panel selection

To identify the diagnostic biomarker panels, a multi-step pipeline was employed. First, the individual discriminatory power of each metabolite was evaluated by calculating the area under the receiver operating characteristic (ROC) curve (AUC) for each metabolite as a single predictor, using the pROC package (v1.18.0)^[23]^. Metabolites with an AUC greater than 0.7 were considered to have strong individual classification ability and were retained as initial candidates. For exploratory biomarker screening, both parametric (Student's *t*-test) and non-parametric (Wilcoxon rank-sum test) comparisons were examined; metabolites meeting *P* < 0.05 in either analysis were carried forward as candidate biomarkers for subsequent combinatorial modeling.

Subsequently, a systematic combinatorial analysis was performed by constructing logistic regression models for all possible combinations of candidate biomarkers. To manage computational load for larger sets of features, combinations were randomly sampled down to a maximum of 10000 for each value of K. Top-ranked combinations were further evaluated using 10-fold cross-validation to provide an internal estimate of classification performance^[24]^; however, because feature screening and panel selection preceded cross-validation, these estimates may remain optimistic. To enrich for biomarkers relevant to endogenous physiology, we prioritized metabolites known to participate in established human metabolic pathways. Compounds with clear xenobiotic signatures or limited physiological relevance were excluded to improve interpretability.

### Statistical analysis

All statistical analyses were performed using R software (v4.2.0; R Foundation for Statistical Computing, Vienna, Austria). For the initial biomarker screening, both the two-tailed Student's *t*-test and the Wilcoxon rank-sum test were applied to each metabolite, and metabolites significant in either test (*P* < 0.05) were retained (see Biomarker panel selection). For all subsequent group comparisons presented in the figures (*e.g.*, boxplots of individual metabolite abundances), the two-tailed Student's *t*-test was used, as indicated in the figure legends.

## Results

### Seminal plasma metabolomic signatures distinguished NOA from OA

**Figure 1:**
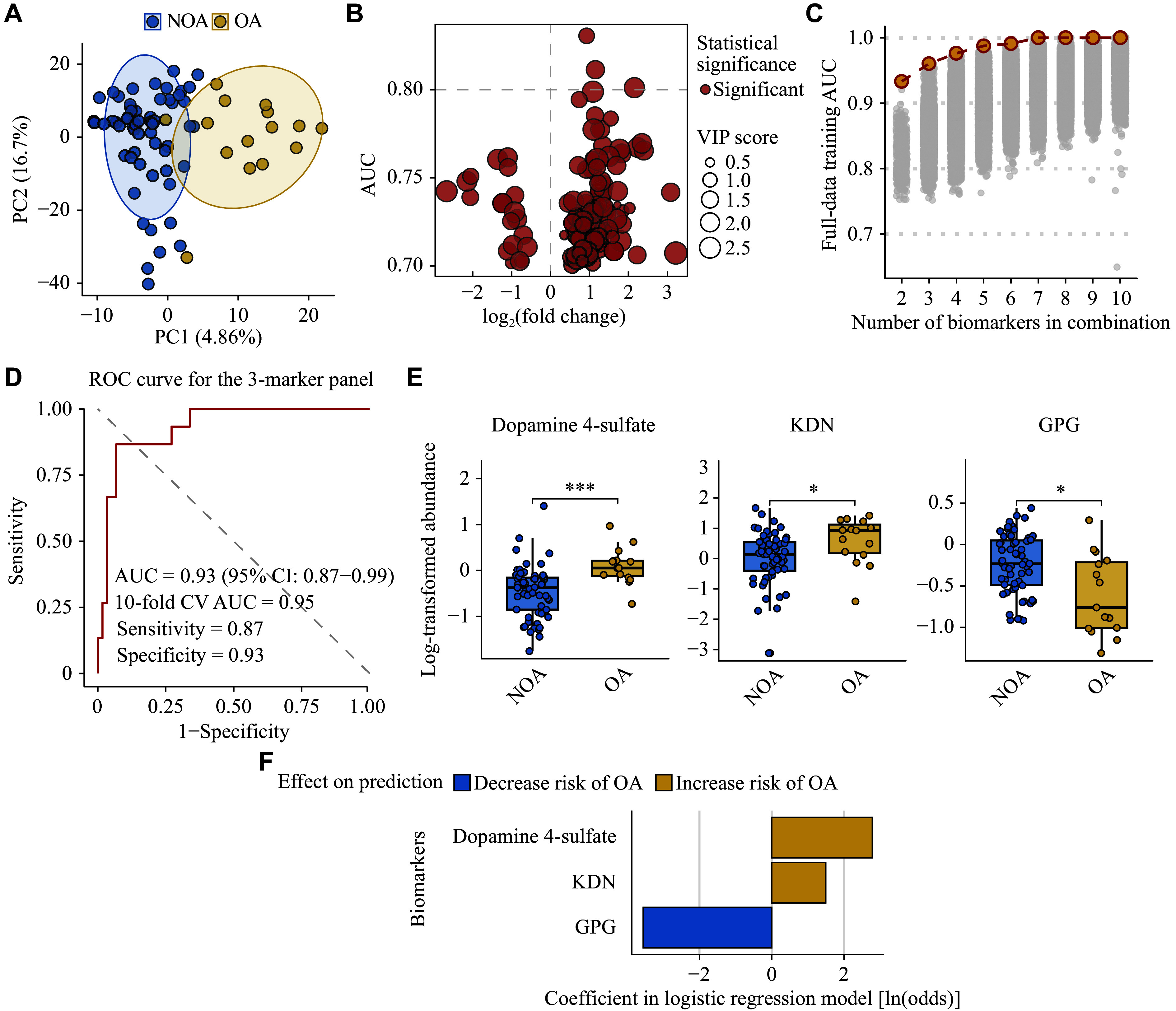
Identification of metabolic biomarkers distinguishing NOA from OA in human seminal plasma. A: OPLS-DA score plot of seminal plasma metabolomic profiles from patients with NOA (blue, *n* = 59) and OA (yellow, *n* = 15). Ellipses indicate the 95% confidence interval for each group. B: The bubble plot of metabolites with high individual discriminatory power (AUC > 0.7). The x-axis represents the log_2_(fold change) (NOA *vs.* OA), and the y-axis represents the AUC. Bubble size corresponds to the variable importance in projection (VIP) score from OPLS-DA, and red color indicates statistical significance (*P* < 0.05). C: Performance of logistic regression models with different numbers of biomarkers. Each gray dot represents a unique biomarker combination, while the red-circled dots indicate the combination with the highest full-data training AUC for that number of markers. D: Receiver operating characteristic (ROC) curve for the selected three-marker panel, with AUC, sensitivity, specificity, and 10-fold CV AUC indicated. E: Boxplots showing the log-transformed abundance of the three individual biomarkers in the NOA and OA groups. Boxplots display median and interquartile ranges, with raw data overlaid as jitter points. Statistical significance was assessed by Student's *t*-test. ^*^P < 0.05 and ^***^P < 0.001. F: Logistic regression coefficients for each biomarker, indicating their contribution to OA/NOA classification. Coefficients are natural log-odds. Abbreviations: AUC, area under the curve; CI, confidence interval; CV, cross-validation; GPG, glycerophosphoglycerol; KDN, 3-deoxy-D-glycero-D-galacto-2-nonulosonic acid; NOA, non-obstructive azoospermia; OA, obstructive azoospermia; OPLS-DA, orthogonal partial least squares-discriminant analysis.

Building upon our previous work that established a distinct metabolic signature for azoospermia, the present study aimed to dissect the metabolic heterogeneity within azoospermia^[20]^. Clinical characteristics of the patients in each disease subtype are provided in ***Supplementary Table 1***. To investigate the metabolic alterations underlying spermatogenic failure, we first performed a comparative metabolomic analysis on seminal plasma from patients with NOA and OA. The OA group, which generally retains spermatogenic activity despite reproductive tract obstruction, served as a clinical comparison group. OPLS-DA of the metabolomic data revealed a distinct separation between the NOA and OA groups (***Fig. 1A***), indicating a systematic difference in the overall metabolic profiles of their seminal plasma.

To identify potential biomarkers, we calculated the AUC for each metabolite and selected those with an AUC > 0.7 as candidate markers. We further annotated these candidates based on their statistical significance (*P* < 0.05), fold change, and variable importance in projection score. The discriminatory power and statistical significance of these candidate metabolites are visualized in a bubble plot (***Fig. 1B***).

To develop a clinically applicable and parsimonious candidate biomarker panel, we employed a systematic pipeline to select a high-performing biomarker panel. By evaluating the performance of logistic regression models built from different combinations of these metabolites, we observed that the predictive accuracy (full-data training AUC) rapidly increased and plateaued with a small number of markers (***Fig. 1C***). ***Supplementary Table 2*** contains the complete list of all analyzed metabolites, along with fold changes, *P* values, and AUC values from ROC analysis. While the combinations with more markers could achieve marginally higher AUCs, we prioritized the balance between model simplicity and predictive power. From the initial pool of high-performing candidates, we then prioritized biomarker panels composed of metabolites primarily reflecting endogenous metabolic pathways. This was done to enhance biological interpretability, focusing on compounds central to cellular processes rather than those likely originating from direct dietary or environmental exposure. Consequently, we selected the candidate three-marker panel, which comprised dopamine 4-sulfate, 3-deoxy-D-glycero-D-galacto-2-nonulosonic acid (KDN), and glycerophosphoglycerol (GPG), as it demonstrated discriminatory performance without unnecessary complexity.

The ROC curve analysis for this three-marker panel yielded an AUC of 0.93 (95% confidence interval [CI]: 0.87–0.99), with an apparent sensitivity of 86.7% and a specificity of 93.2% in the full analysis dataset at the optimal cutoff. Internal 10-fold cross-validation yielded an AUC of 0.95, although this estimate requires confirmation in an independent cohort (***Fig. 1D***). Analysis of individual markers revealed that dopamine 4-sulfate and KDN levels were significantly elevated in the OA group, where spermatogenesis is active, compared with the NOA group. Conversely, GPG levels were significantly higher in the NOA group than in the OA group (***Fig. 1E***). Additionally, the logistic regression model coefficients demonstrated the directional contribution and relative weight of each of the three metabolites in the predictive model (***Fig. 1F***).

### Comparison of seminal plasma metabolomic characteristics between SCO and non-SCO type NOA

**Figure 2:**
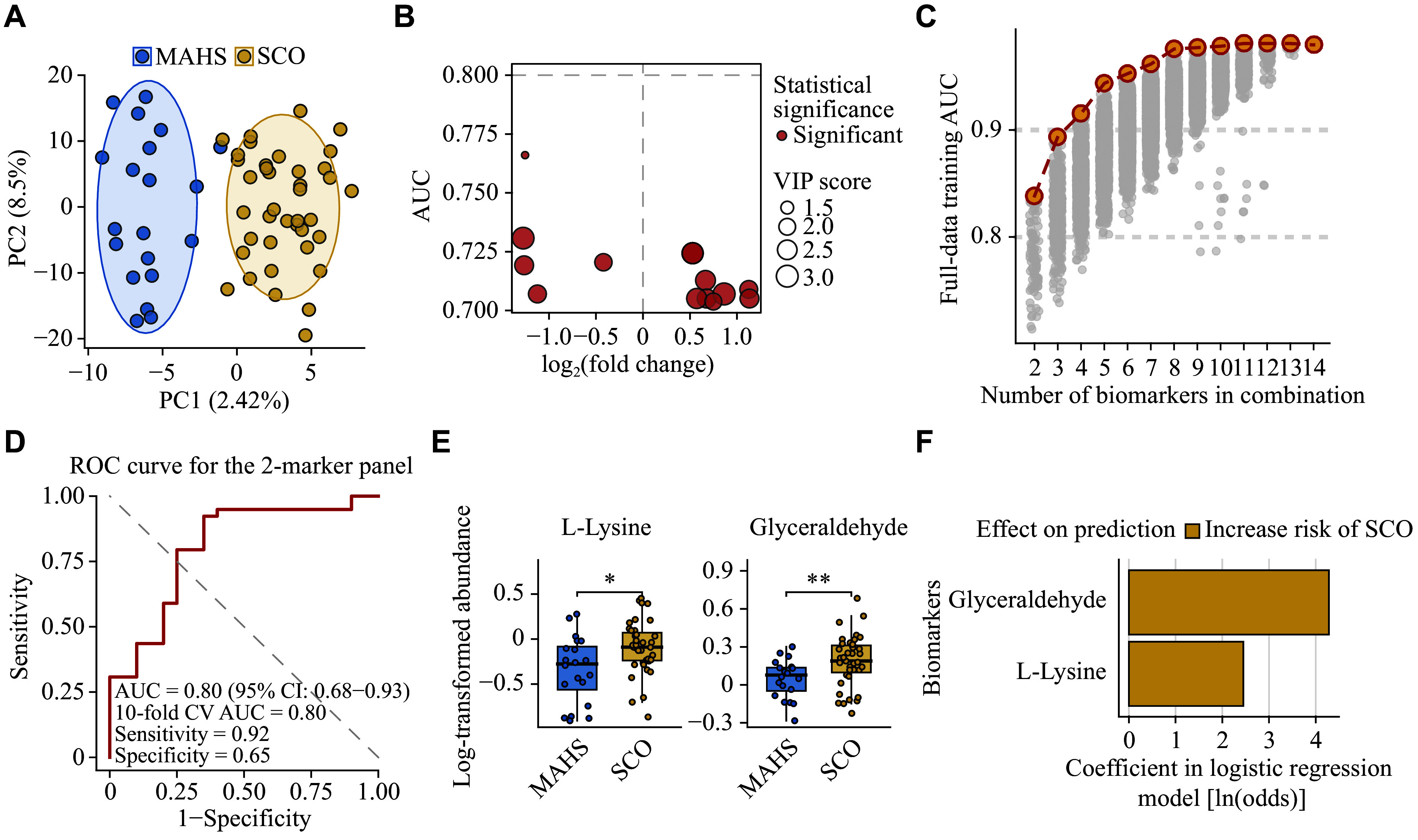
Identification of metabolic biomarkers distinguishing MAHS from SCO in human seminal plasma. A: OPLS-DA score plot of seminal plasma metabolomic profiles from patients with MAHS (blue, *n* = 20) and SCO (yellow, *n* = 39). Ellipses indicate the 95% confidence interval for each group. B: Bubble plot of metabolites with high individual discriminatory power (AUC > 0.7). The x-axis represents the log_2_(fold change) (MAHS *vs.* SCO), and the y-axis represents the AUC. Bubble size corresponds to the variable importance in projection (VIP) score from OPLS-DA, and red color indicates statistical significance (*P* < 0.05). C: Performance of logistic regression models with different numbers of biomarkers. Each gray dot represents a unique biomarker combination, while the red-circled dots indicate the combination with the highest full-data training AUC for that number of markers. D: Receiver operating characteristic (ROC) curve for the optimal two-marker panel, with AUC, sensitivity, specificity, and 10-fold CV AUC indicated. E: Boxplots showing the log-transformed abundance of the two individual biomarkers in the MAHS and SCO groups. Boxplots display median and interquartile ranges, with raw data overlaid as jitter points. Statistical significance was assessed by Student's *t*-test. ^*^P < 0.05 and ^**^P < 0.01. F: Logistic regression coefficients for each biomarker, indicating their contribution to MAHS/SCO classification. Coefficients are natural log-odds. Abbreviations: AUC, area under the curve; CI, confidence interval; CV, cross-validation; MAHS, maturation arrest and hypospermatogenesis; NOA, non-obstructive azoospermia; OA, obstructive azoospermia; OPLS-DA, orthogonal partial least squares-discriminant analysis; SCO, Sertoli cell-only syndrome.

To further dissect the metabolic signatures related to varying degrees of spermatogenic impairment, we stratified NOA patients based on their testicular histology. This distinction is clinically critical, as the complete absence of germ cells in SCO offers a significantly poorer prognosis for sperm retrieval compared with cases that retain germ cells^[25]^. We therefore conducted a comparative metabolomic analysis on seminal plasma from SCO patients against a combined group of patients with MA and HS, hereafter referred to as the MAHS group. OPLS-DA revealed a clear metabolic distinction between the SCO and MAHS groups, suggesting that these distinct histological patterns of spermatogenic failure have unique metabolic footprints (***Fig. 2A***). A bubble plot of 14 metabolites with high individual discriminatory power and statistical significance highlighted several promising candidates for differentiating these two conditions (***Fig. 2B***). A comprehensive list of all analyzed metabolites is detailed in ***Supplementary Table 3***. We systematically evaluated all possible combinations of these candidate metabolites for their diagnostic performance (***Fig. 2C***). Prioritizing model parsimony for potential clinical utility, we focused on candidates composed of metabolites involved in endogenous metabolic pathways. The selected two-marker panel, consisting of L-lysine and glyceraldehyde, achieved an AUC of 0.80 (95% CI: 0.68–0.93), with an apparent sensitivity of 92.3% and a specificity of 65.0% in the full analysis dataset. Internal 10-fold cross-validation yielded an AUC of 0.80 (***Fig. 2D***).

Analysis of the individual metabolites showed that both L-lysine and glyceraldehyde levels were significantly elevated in the SCO group compared with the MAHS group (***Fig. 2E***). Additionally, the logistic regression model coefficients demonstrated this finding, showing that higher levels of both L-lysine and glyceraldehyde increased the likelihood of a SCO classification (***Fig. 2F***). These results identify a candidate two-marker signature with potential for distinguishing SCO from other major histological subtypes of NOA, offering insights into the metabolic pathways that may be related to the severity of spermatogenic failure.

### Comparing seminal plasma metabolomic profiles between HS and MA

**Figure 3:**
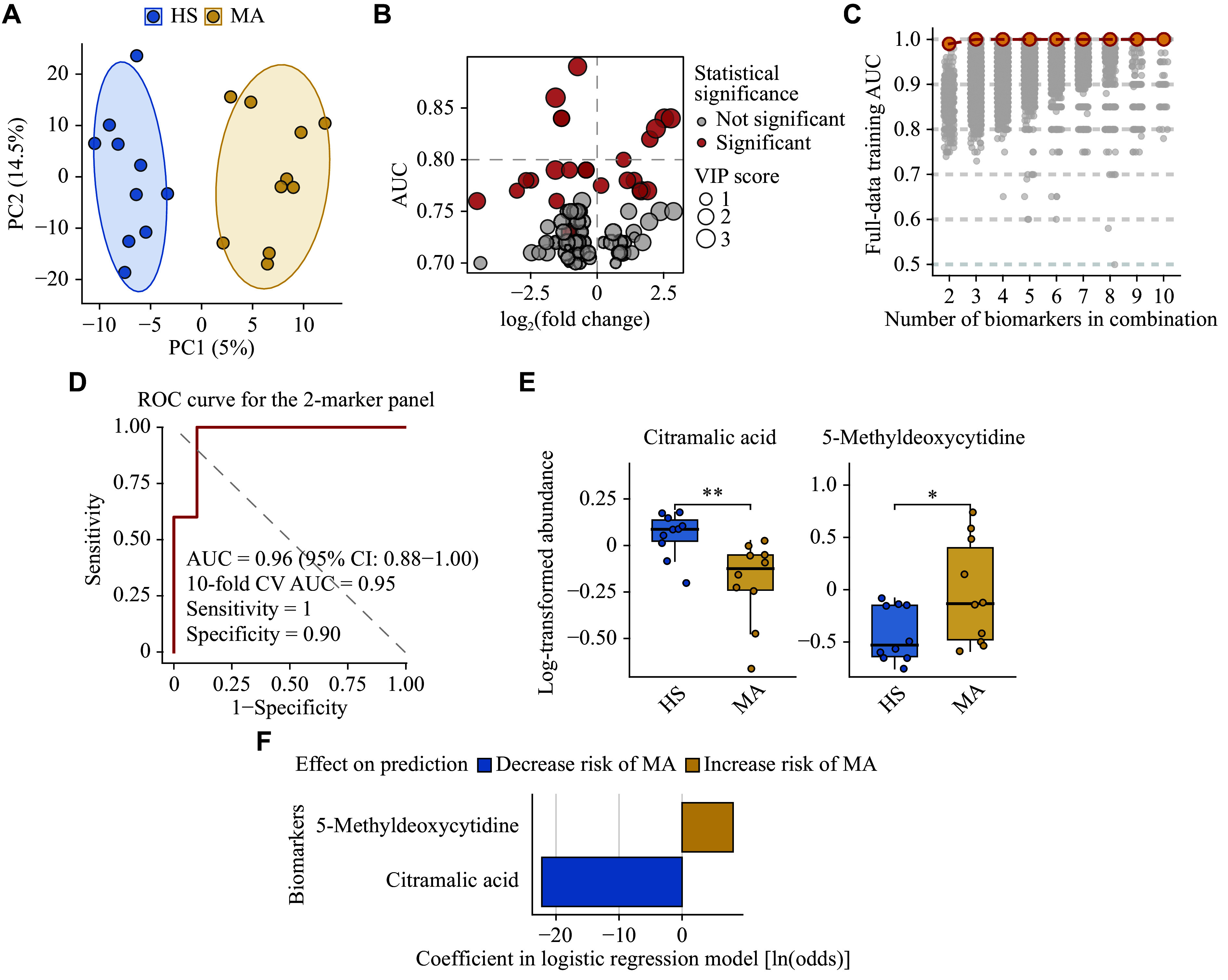
Identification of metabolic biomarkers distinguishing HS from MA in human seminal plasma. A: OPLS-DA score plot of seminal plasma metabolomic profiles from patients with HS (blue, *n* = 10) and MA (yellow, *n* = 10). Ellipses indicate the 95% confidence interval for each group. B: Bubble plot of metabolites with high individual discriminatory power (AUC > 0.7). The x-axis represents the log_2_(fold change) (HS *vs.* MA), and the y-axis represents the AUC. Bubble size corresponds to the variable importance in projection (VIP) score from OPLS-DA, and red color indicates statistical significance (*P* < 0.05). C: Performance of logistic regression models with different numbers of biomarkers. Each gray dot represents a unique biomarker combination, while the red-circled dots indicate the combination with the highest full-data training AUC for that number of markers. D: Receiver operating characteristic (ROC) curve for the selected two-marker panel, with AUC, sensitivity, specificity, and 10-fold CV AUC indicated. E: Boxplots showing the log-transformed abundance of the two individual biomarkers in the HS and MA groups. Boxplots display median and interquartile ranges, with raw data overlaid as jitter points. Statistical significance was assessed by Student's *t*-test. ^*^P < 0.05 and ^**^P < 0.01. F: Logistic regression coefficients for each biomarker, indicating their contribution to HS/MA classification. Coefficients are natural log-odds. Abbreviations: AUC, area under the curve; CI, confidence interval; CV, cross-validation; HS, hypospermatogenesis; MA, maturation arrest; OPLS-DA, orthogonal partial least squares-discriminant analysis.

Building on the previous findings, we sought to determine if the subtypes within the broader MAHS group, specifically HS and MA, could also be metabolically distinguished. HS often implies a more favorable prognosis for sperm retrieval than MA, where spermatogenesis is blocked at a specific meiotic or post-meiotic stage^[4,26]^. OPLS-DA revealed a distinct separation of seminal plasma metabolic profiles between the two groups, indicating that HS and MA, despite both being forms of incomplete spermatogenesis, possess unique metabolic signatures (***Fig. 3A***). A bubble plot highlighted a total of 26 metabolites with high individual discriminatory power and statistical significance, all of which were promising candidates for differentiating these two conditions (***Fig. 3B***). ***Supplementary Table 4*** provides a complete breakdown of all metabolites. Our systematic evaluation of biomarker combinations indicated that exceptional predictive performance could be achieved with a minimal number of markers, as the training AUC peaked at nearly 1.0 with only a two-marker panel (***Fig. 3C***). Based on these findings, we selected the most parsimonious and effective two-marker panel, consisting of citramalic acid and 5-methyldeoxycytidine. ROC curve analysis demonstrated the high classification performance of the two-marker panel, yielding an AUC of 0.96 (95% CI: 0.88–1.00), with an apparent sensitivity of 100% and a specificity of 90.0% in the full analysis dataset at the optimal cutoff. Internal 10-fold cross-validation produced a CV AUC of 0.95 (***Fig. 3D***). Given the small sample size, this high apparent classification performance should be interpreted cautiously and requires independent validation.

Analysis of the individual markers revealed opposing trends between the two conditions. Citramalic acid levels were significantly higher in the HS group, whereas 5-methyldeoxycytidine levels were significantly higher in the MA group (***Fig. 3E***). The elevation of 5-methyldeoxycytidine, a methylated deoxycytidine derivative associated with DNA methylation metabolism, is noteworthy and may reflect altered nucleic acid turnover or epigenetic regulation in MA, a known contributor to meiotic arrest^[27]^. This was reflected in the logistic regression model coefficients, where higher citramalic acid levels predicted an HS classification, and higher 5-methyldeoxycytidine levels predicted an MA classification (***Fig. 3F***). These findings identify a promising candidate two-marker panel for differentiating HS from MA, although independent validation is required.

### Cross-species integrative analyses elucidated the physiological underpinnings of biomarkers for azoospermia

To explore the potential biological context of the metabolic disruptions observed in NOA, we performed a comparative analysis between our human seminal plasma data and a previously established metabolomic dataset from purified mouse testicular cell populations^[20]^. It is important to note that this cross-species analysis is exploratory and intended to generate hypotheses about general metabolic pathways affected by spermatogenic arrest. This dataset provides metabolic profiles of specific testicular cell populations: pachytene spermatocytes, round spermatids (RS), elongating spermatids (ES), and Sertoli cells, enabling the linkage of seminal plasma alterations to specific cellular processes and developmental stages.

**Figure 4:**
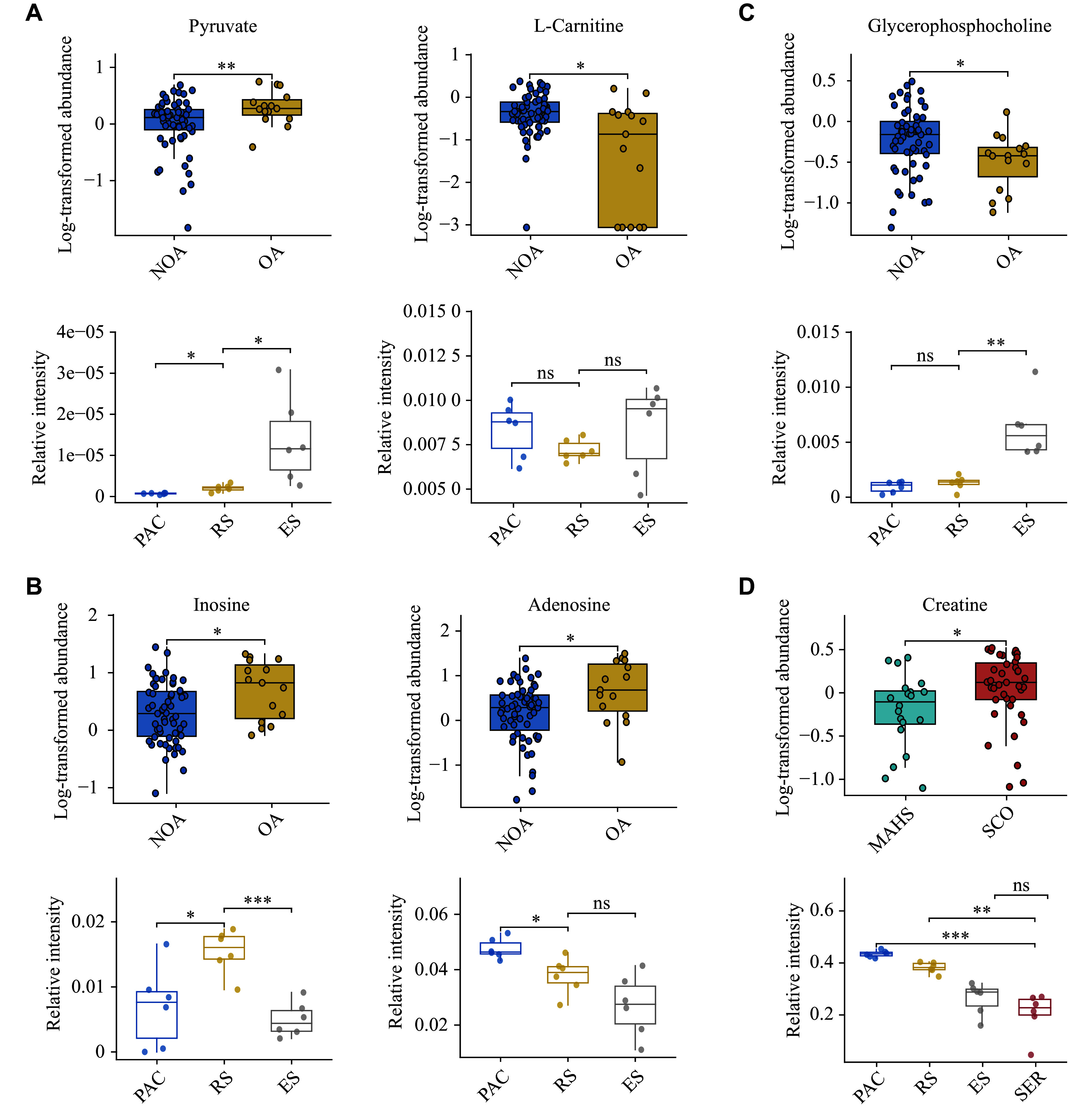
Comparison of key metabolite abundances in human seminal plasma and mouse testicular cell types. The abundance of pyruvate and L-carnitine (A), adenosine and inosine (B), GPC (C), and creatine (D) in human seminal plasma and mouse testes. Human seminal plasma levels are compared between NOA and OA (A–C) and between MAHS and SCO (D). Mouse testicular levels are shown for PAC, RS, ES (A–C), and SER (D), with *n* = 6 per cell type. Boxplots display median and interquartile ranges, with raw data overlaid as jitter points. Statistical significance is indicated as follows: ^*^P < 0.05, ^**^P < 0.01, and ^***^P < 0.001 by Student's *t*-test. Abbreviations: ES, elongating spermatids; GPC, glycerophosphocholine; MAHS, maturation arrest and hypospermatogenesis; NOA, non-obstructive azoospermia; ns, not significant; OA, obstructive azoospermia; PAC, pachytene spermatocytes; RS, round spermatids; SCO, Sertoli cell-only syndrome; SER, Sertoli cells.

Beyond the biomarkers we presented, several classical metabolites involved in energy and membrane metabolism displayed significant alterations in NOA compared with OA (***Fig. 4A***). Pyruvate, a critical fuel for the tricarboxylic acid cycle, was significantly reduced in the seminal plasma of NOA patients^[28]^. Our mouse data revealed that pyruvate levels progressively increase through spermatogenesis, peaking in the ES to fuel the highly energy-demanding process of spermiogenesis. The absence of late-stage germ cells in NOA may contribute to the lower pyruvate levels observed in seminal plasma. Conversely, L-carnitine, which transports fatty acids for mitochondrial β-oxidation, was significantly elevated in NOA. While our mouse data revealed its high abundance and importance in all germ cells, its higher abundance in NOA seminal plasma may partly reflect reduced utilization associated with the absence of sperm. L-carnitine is crucial for sperm maturation in the epididymis; in NOA, where no sperm reach the epididymis, this factor may accumulate^[29]^.

We also observed significant reductions in the purine metabolites inosine and adenosine in NOA seminal plasma (***Fig. 4B***). Mouse data revealed that both metabolites significantly declined during the transition from RS to ES, indicating a tightly regulated role for purine metabolism during this critical window of post-meiotic differentiation. This stage-specific enrichment may support nucleic acid turnover, redox regulation, or nucleotide recycling during differentiation^[30–31]^. The depletion of these purines in NOA thus aligns with either an arrest before RS in MAHS or the complete absence of germ cells in SCO.

Glycerophosphocholine (GPC), a key metabolite in membrane remodeling^[32–33]^, was higher in NOA than in OA seminal plasma (***Fig. 4C***). Mouse data revealed GPC levels peaked in ES, consistent with the extensive membrane reorganization during spermiogenesis. Similar to L-carnitine, the higher GPC levels in NOA may partly reflect altered utilization or transport within the male reproductive tract.

A key observation from NOA subtype comparisons was that creatine levels were significantly higher in the seminal plasma of SCO patients than in those of MA and HS patients (***Fig. 4D***). Consistently, data from mouse testis indicated that creatine is predominantly detected in germ cells, while Sertoli cells exhibited relatively low levels of creatine-associated metabolites. Sertoli cells are thought to supply creatine to developing germ cells, which act as a metabolic sink^[34–35]^. In MAHS samples with partially retained germ cells, this utilization may be preserved to some extent. Conversely, complete germ cell loss in SCO disrupts this process, potentially resulting in creatine accumulation in testicular fluid and, subsequently, in seminal plasma. While the exact source and intercellular trafficking of creatine in the testis remain to be fully clarified, the observed pattern supports a link between germ cell depletion and altered creatine dynamics in NOA.

## Discussion

The present study suggests that seminal plasma metabolomics may enable the identification of candidate biomarker panels for non-invasive stratification of azoospermia subtypes. While our comparison between OA and NOA provides a valuable metabolic benchmark for spermatogenic failure, its direct clinical impact is limited. The clinical promise lies in distinguishing NOA subtypes, offering a potential tool to guide patient counseling and pre-testicular sperm extraction treatment strategies.

A data-driven outlier removal step, guided by PCA diagnostics, was implemented to improve model stability. Although PCA-based outlier exclusion was applied according to prespecified statistical thresholds, the exclusion of participants without identifiable clinical abnormalities may introduce selection bias and should therefore be considered when interpreting the model performance. Our analytical approach began with OPLS-DA, which confirmed a significant metabolic divergence between patient groups. While systematic metabolic differences exist at a population level, the inherent biological variability and complexity are too high for a single linear model to serve as a high-precision diagnostic tool for individual patients. The primary value of our OPLS-DA models lies not in classification but in exploration and hypothesis generation, which is a recognized strength of such exploratory methods^[36]^.

To develop more parsimonious multimetabolite classifiers, we implemented a multi-step biomarker discovery pipeline. This strategy included AUC-based selection of candidate markers, statistical filtering, combinatorial modeling, and internal validation *via* 10-fold cross-verification. The results underscore the importance of combination-based biomarkers over single-metabolite predictors, particularly for complex clinical phenotypes^[37]^.

Our cross-species comparison using metabolomic profiles from normal mouse testicular cells was exploratory, aiming to provide a biological context for the human seminal plasma biomarkers. However, key metabolites from our human diagnostic panels were not consistently detected in mice, likely due to species-specific differences and the complex, multi-organ origin of seminal plasma. Therefore, these cross-species findings should be viewed as hypothesis-generating rather than confirmatory, and future studies using human testicular tissue are needed for further validation.

Despite these promising results, several limitations should be noted. Stratification into histological subgroups, combined with the exclusion of six outliers, resulted in small sample sizes, particularly in the HS versus MA comparison. The near-perfect AUC observed in this subgroup likely reflects overfitting and should be interpreted with caution. Class imbalance in other comparisons, such as NOA versus OA, may also introduce bias despite our mitigation efforts. Additionally, although a strict multiple-testing correction was not applied during exploratory screening, the selected multi-marker panels were further evaluated using internal cross-validation. Nevertheless, the possibility of false-positive feature selection and optimistic performance estimates cannot be excluded. Most critically, all of our biomarker panels have only undergone internal validation *via* 10-fold cross-validation. Because biomarker screening and panel selection were performed before cross-validation, the internal cross-validation estimates may remain optimistic and should not be interpreted as unbiased estimates of performance in independent populations. This procedure, while useful, is no substitute for validation on an independent, external dataset. Consequently, our findings should be considered exploratory and hypothesis-generating, and the proposed panels are not yet ready for clinical application. Their true diagnostic value is entirely contingent on successful validation in future large, multicenter studies. Finally, the cross-sectional design of the present study establishes strong associations but cannot determine causality. It remains to be determined whether the observed metabolic dysregulation is a cause or a consequence of spermatogenic arrest.

In summary, the present study demonstrates the feasibility and potential of seminal plasma metabolomics, combined with systematic model development, for stratifying azoospermia subtypes. These findings support further development of non-invasive biomarker approaches for pre-biopsy assessment and patient stratification.

## Additional information

The online version contains supplementary materials available at http://www.jbr-pub.org.cn/article/doi/10.7555/JBR.39.20250294.
