# Supplementary material for: Stratified profiling of azoospermia: Differentiating histological subtypes with seminal plasma metabolic signatures
Source: J Biomed Res. 2026 Jul 28;40(4):425–35. doi: 10.7555/JBR.39.20250294 (PMC13438305; doi:10.7555/JBR.39.20250294)
Supplement: Supplementary file 1 [file jbr-40-4-425-S1.pdf]

Available online at www.jbr-pub.org.cn
Open Access at PubMed Central
Journal of Biomedical Research, 2026 40(4): 1–1
Supplementary Data
JBR

Stratified profiling of azoospermia: Differentiating histological subtypes with seminal plasma metabolic signatures

Jiachen Wang[1,△], Tianyi Song[1,△], Hongqi Fan[2,△], Mengyuan Zhu[3], Ying Yao[1], Laihua Li[1], Mingxi Liu[1], Minjian Chen[3], Jiahao Sha[1], Xiaoyu Yang[4,✉], Yan Yuan[1,✉]

[1]*State Key Laboratory of Reproductive Medicine and Offspring Health, Nanjing Medical University, Nanjing, Jiangsu 211166, China;*
[2]*Department of Endocrinology, the First Affiliated Hospital of Nanjing Medical University, Nanjing Medical University, Nanjing, Jiangsu 210029, China;*
[3]*Key Laboratory of Modern Toxicology of Ministry of Education, School of Public Health, Nanjing Medical University, Nanjing, Jiangsu 211166, China;*
[4]*State Key Laboratory of Reproductive Medicine and Offspring Health, Clinical Center of Reproductive Medicine, the First Affiliated Hospital of Nanjing Medical University, Nanjing, Jiangsu 210029, China.*

Supplementary Table 1 **Summary of baseline clinical and demographic characteristics for azoospermia subgroups based on samples with available clinical data in this study**

Variants	SCO	MA	HS	OA
Number of samples	39	11	12	18
Age [years, mean (range)]	30.0 (23.0–44.0)	30.0 (26.0–34.0)	30.0 (26.0–34.0)	31.0 (26.0–38.0)
FSH (IU/L, mean)	20.6	12.3	20.5	5.0
Sperm concentration (million/mL, mean)	0	0	0	0
Left testicular volume (mL, mean)	5.5	11.0	8.2	15.0
Right testicular volume (mL, mean)	5.2	11.0	6.7	15.0
BMI (kg/m^2, mean)	25.2	25.3	25.4	24.6

Samples shown represent the full clinically evaluable cohort. Statistical outliers excluded from metabolomics modeling are still included in this baseline table. Abbreviations: BMI, body mass index; FSH, follicle-stimulating hormone; HS, hypospermatogenesis; MA, maturation arrest; OA, obstructive azoospermia; SCO, Sertoli cell-only syndrome.

Results from comprehensive statistical analyses of seminal plasma metabolites, including comparisons of non-obstructive azoospermia (NOA) *vs.* obstructive azoospermia (OA) patients, Sertoli cell-only syndrome (SCO) *vs.* other NOA subtypes (MAHS group), and hypospermatogenesis (HS) *vs.* maturation arrest (MA) patients, are shown in ***Supplementary Tables 2–4*** (available online), respectively.

△These authors contributed equally to this work.
✉Corresponding authors: Xiaoyu Yang, E-mail: yxy1921@163.com, ORCID: 0000-0002-6411-8935; Yan Yuan, E-mail: yuanyan@njmu.edu.cn, ORCID: 0000-0002-6773-1188.

Received: 12 July 2025; Revised: 30 August 2025; Accepted: 02 September 2025; Available online: 11 September 2025; Published date: 28 July 2026

CLC number: R698.2, Document code: A

The authors reported no conflicts of interest.

This is an open access article under the Creative Commons Attribution (CC BY 4.0) license, which permits others to distribute, remix, adapt and build upon this work, for commercial use, provided the original work is properly cited.

© 2026 by Journal of Biomedical Research.

https://doi.org/10.7555/JBR.39.20250294
